# Fe protein docking transduces conformational changes to MoFe nitrogenase active site in a nucleotide-dependent manner

**DOI:** 10.1038/s42004-023-01046-6

**Published:** 2023-11-18

**Authors:** Monika Tokmina-Lukaszewska, Qi Huang, Luke Berry, Hayden Kallas, John W. Peters, Lance C. Seefeldt, Simone Raugei, Brian Bothner

**Affiliations:** 1 Department of Chemistry and Biochemistry, Montana State University, Bozeman, MT USA; 2 Physical and Computational Sciences Directorate, Pacific Northwest National Laboratory, Richland, WA USA; 3 Department of Chemistry and Biochemistry, Utah State University, Logan, UT USA; 4 Institute of Biological Chemistry, The University of Oklahoma, Norman, OK USA

**Keywords:** Biophysical chemistry, Molecular modelling, Enzyme mechanisms, Metalloproteins, Mass spectrometry

## Abstract

The reduction of dinitrogen to ammonia catalyzed by nitrogenase involves a complex series of events, including ATP hydrolysis, electron transfer, and activation of metal clusters for N_2_ reduction. Early evidence shows that an essential part of the mechanism involves transducing information between the nitrogenase component proteins through conformational dynamics. Here, millisecond time-resolved hydrogen-deuterium exchange mass spectrometry was used to unravel peptide-level protein motion on the time scale of catalysis of Mo-dependent nitrogenase from *Azotobacter vinelandii*. Normal mode analysis calculations complemented this data, providing insights into the specific signal transduction pathways that relay information across protein interfaces at distances spanning 100 Å. Together, these results show that conformational changes induced by protein docking are rapidly transduced to the active site, suggesting a specific mechanism for activating the metal cofactor in the enzyme active site.

## Abstract (web summary)

The reduction of dinitrogen to ammonia catalyzed by nitrogenase involves long-range conformational changes within the enzyme complex, however, direct biophysical evidence of communication between the Fe protein and the MoFe protein is lacking. Here, the authors combine millisecond time-resolved hydrogen-deuterium exchange mass spectrometry and normal mode analysis, revealing molecular-level insights into how the Fe protein alters the stability and dynamics of the MoFe protein near the active site in a nucleotide-dependent manner.

## Introduction

**Fig. 1:**
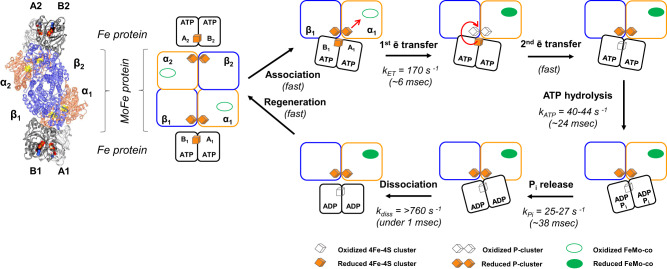
The Mo-dependent nitrogenase complex composition and catalytic cycle. The structural model is MoFe protein:Fe protein 1:2 (PDB ID: 4WZB). In this structure, Fe protein is bound by a non-hydrolyzable analog of ATP (β,γ-methylene MgATP) shown as space-filling atoms. The scheme shows the steps in the catalytic cycle based on ½ of the nitrogenase complex. The nitrogenase Fe protein cycle involves transient associations between the reduced, MgATP-bound Fe protein and the MoFe protein. It includes electron transfer, ATP hydrolysis, the release of P_i_, and dissociation of the oxidized, MgADP-bound Fe protein from MoFe protein. To complete the cycle, Fe protein needs to be re-charged ([4Fe-4S] cluster is reduced) by accepting electrons from an available electron donor. Note that the orientation of the Fe protein depends on nucleotide type. Also, the position of [4Fe-4S] cluster in the MgATP-bound Fe protein is closer to the P-cluster than in the case of the MgADP-bound form. Experimental time points were designed to report pre-steady-state steps in the catalytic cycle. Provided rate constants for the first electron transfer, ATP hydrolysis, P_i_ release and nitrogenase complex dissociation are from Yang et al. ^11^.

The availability of fixed nitrogen limits the growth of plant life on Earth, and the current practices for applying nitrogenous fertilizers can compromise soil health. As a result, research on biological nitrogen fixation is of high agronomic, economic, and environmental significance^1^. Nitrogenase is responsible for biological nitrogen fixation, catalyzing the reduction of N_2_ to NH_3_. There are three evolutionarily related forms of the enzyme, which differ slightly in the composition of their metal-containing prosthetic groups^2–10^. Mo-dependent nitrogenase is the most common and well-studied form. This enzymatic complex has two separable components: the Fe and MoFe proteins. Catalysis is a multi-step process where the rates of the individual steps range from microseconds to milliseconds, making the Mo-dependent nitrogenase relatively slow-turnover enzyme^11,12^. There is ample evidence from studies over decades that N_2_ reduction involves association and dissociation of the Fe protein and MoFe protein, during which the P-clusters accept electrons from the Fe protein as an intermediate in the transfer of electrons to the FeMo-cofactors (FeMo-co) active sites where N_2_ is reduced to NH_3_ (Fig. 1)^2,11,13–23^. This Fe protein cycle must occur eight times to fulfill the minimum stoichiometry (Eq. 1), where the high energetic cost is attributed to the energy necessary for N_2_ triple bond activation.

1 $${{{{{{\rm{N}}}}}}}_{2}+8{{{{{{\rm{e}}}}}}}^{-}+8{{{{{{\rm{H}}}}}}}^{+}+16{{{{{\rm{MgATP}}}}}}\to 2{{{{{{\rm{NH}}}}}}}_{3}+{{{{{{\rm{H}}}}}}}_{2}+16{{{{{\rm{MgADP}}}}}}+16{{{{{{\rm{P}}}}}}}_{{{{{{\rm{i}}}}}}}$$

How MgATP binding and hydrolysis within the Fe protein affects the chemistry at the FeMo-co ~30 Å away remains unclear. Growing evidence indicates long-range conformational changes within the complex are essential to catalysis^12,23–31^. Recent pre-steady-state kinetic studies uncovered evidence that electron transfer at one αβ dimer of the MoFe protein suppresses electron transfer in the other αβ dimer through a half-sites cooperativity mechanism^12^. It was proposed that the half-sites reactivity was imposed through protein conformational changes that allosterically suppress electron transfer in one αβ dimer until it is completed in the other. In support of these conformational controls, graph theory analysis and near-the-equilibrium hydrogen-deuterium exchange (HDX) studies of the mechanical coupling in the nitrogenase complex suggested that large-amplitude correlated motions could enable communications between regions near the active site and the Fe protein within each half and between the two halves of the complex^12,32^. However, direct biophysical evidence of communication between the Fe protein and the FeMo-co on a catalytically relevant timescale is lacking.

This study investigates the solution-phase properties of MoFe protein and MoFe protein:Fe protein using HDX and computationally with normal mode analysis (NMA). HDX and NMA are orthogonal approaches that report on protein dynamics. HDX measures deuterium uptake by the protein backbone, reporting on the stability of the hydrogen bond network and solvent accessibility. HDX adds a temporal component to the dynamics by tracking the exchange rate. NMA describes the dynamics of a protein in thermal equilibrium and allows us to determine the type and magnitude of the correlation between the motion amino acids in the protein (or protein complex in the case of MoFe protein:Fe protein). In summary, NMA provides information on the magnitude and direction of motion, while HDX provides information on the time scale of the dynamics. Herein, we describe millisecond time-resolved HDX-MS studies correlating with pre-steady-state catalytic processes. Coupled with computational simulations, we provide molecular-level insights into how Fe protein alters the stability and dynamics of residues in the first coordination sphere of the FeMo-co in a nucleotide-dependent manner.

## Results and discussion

### Fe protein binding alters FeMo-co protein environment on milisecond to minutes time scale

**Fig. 2:**
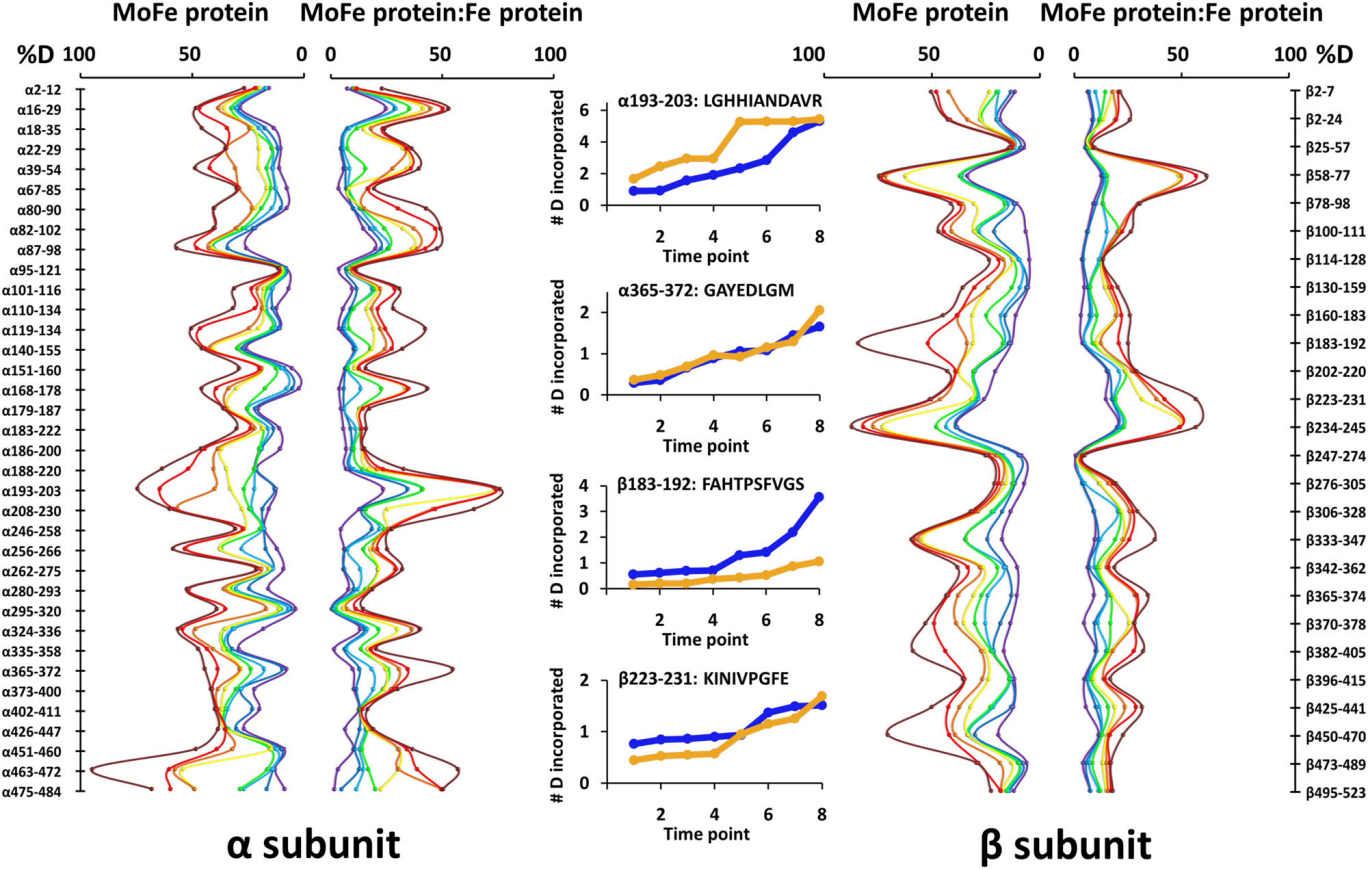
Temporal deuterium incorporation into MoFe protein in the presence and absence of Fe proteins. The butterfly plots (left and right) show the percent deuterium incorporation (%D) at 10 msec (purple), 30 msec (blue), 60 msec (cyan), 80 msec (green), 2 min (yellow), 4 min (orange), 15 min (red), and 60 min (brown). Peptic peptides are shown from the N to C terminus of MoFe protein α and β subunits on the y axis. Regions of the plot that are not symmetrical indicate changes in peptide stability/dynamics related to Fe protein docking, for example, α193-203 or β183-192. Peptides α365-372 and β223-231 highlight regions that change from locally lower levels of exchange to higher upon Fe protein binding. Error bars have been removed for clarity. Over 95% of the data points have a standard deviation of exchange of <5%. By comparing profiles with the same color, deuterium uptake can be compared between MoFe protein alone and in the presence of Fe proteins. Uptake curves show deuterium incorporation at experimental time points for MoFe protein alone in blue and mixed with Fe protein in orange.

Earlier studies pointed to mechanically mediated, long-range communication between the two halves of the nitrogenase complex at equilibrium^32^. To resolve these communications at a molecular level, HDX-MS experiments were conducted both during steady-state turnover (seconds to minutes time scale) and pre-steady-state (quench-flow experiments on the millisecond time scale). Quench-flow experiments allow measuring changes in solvent accessibility and hydrogen bond stability of MoFe protein on a catalytically relevant time scale. Here, HDX-MS was used to measure changes in deuterium uptake in free MoFe protein and the presence of MgATP-bound Fe protein under anaerobic conditions. Pepsin digests of the MoFe protein α and β subunits with a 99% and 100% sequence coverage, respectively. The percent deuterium incorporation (%D) was normalized to the 24-h time point and is reported only for peptides that showed a high confidence deuterium incorporation score in HD Examiner and that were detected in all conditions and time points (37 peptides from the α subunit and 26 peptides from the β subunit can be found in Supplementary Data 1). A global analysis confirmed that Fe protein-dependent changes to the exchange rates in MoFe protein span the entire time course from pre-steady-state to equilibrium conditions. In addition, the magnitude of the changes in exchange is region-specific (Fig. 2). For example, we looked closely at the exchange pattern for the MoFe protein peptide β183-192, which, based on the crystal structure of the nitrogenase Fe protein—MoFe β,γ-methylene MgATP complex (4WZB), makes contact with Fe protein (BLys170, BArg140 and BArg100). Upon Fe protein binding to the MoFe protein, a significant reduction in %D on the minutes time scale is observed. Another example is MoFe protein peptide α191-203, which resides close to FeMo-co, the P-cluster, and the Fe protein Switch I region (AGlu68). For this peptide, a nearly two-fold increase in exchange is observed in the presence of MgATP-bound Fe protein at the first four time points, 10-80 msec, consistent with the early stages of the catalytic cycle (pre-steady-state). MoFe protein peptides α365-372 and β223-231 highlight regions that change from locally lower levels of exchange to higher upon Fe protein binding. The results in Fig. 2 indicate the presence of widely distributed changes to the HDX rate in MoFe protein upon Fe protein binding, spanning the millisecond to hour time regime.

**Fig. 3:**
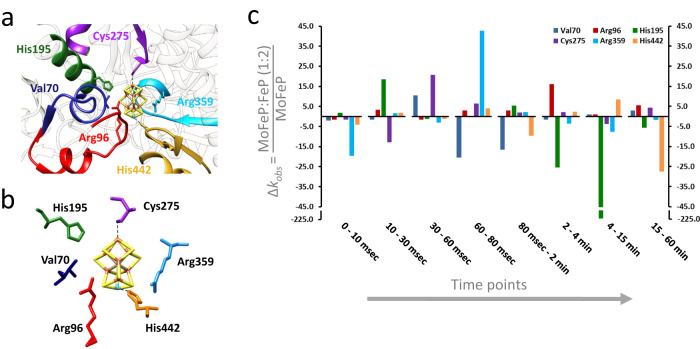
Effect of Fe protein on HDX kinetics in peptides surrounding the MoFe protein active site. **a** Close-up of FeMo-co cluster (PDB ID: 4WZB) and its protein environment (α subunit) containing peptic peptides including the selected residues from the first coordination sphere. **b** FeMo-co cluster and amino acids in the first coordination sphere (PDB ID: 4WZB). **c** Relative change in HDX rate for residues in the first coordination sphere of FeMo-co upon Fe protein binding; negative and positive values indicate a faster change in rate in the MoFe protein alone and the presence of two MgATP-bound Fe proteins, respectively. Order of residues in each time point is as follows: Val70, Arg96, His195, Cys275, Arg359 and His442.

To understand how Fe protein binding alters the environment around FeMo-co, we examined the exchange rate of MoFe protein peptides containing residues in the first coordination sphere of FeMo-co (Fig. 3a, b). HDX shows that MgATP-Fe protein binding causes site-specific, time-dependent changes in protein dynamics in the first coordination sphere of the FeMo-co. For example, in the first 10 msec after Fe protein binding, there was a 20-fold decrease in the exchange rate near Arg359, while other regions experienced insignificant changes (Fig. 3c). At 80 msec (consistent with the end of pre-steady-state) after Fe protein addition, HDX near Arg359 increased by 45-fold, while it decreased by 20-fold near Val70. On the minutes time scale, we also observed Fe protein-dependent changes in the dynamics of the residues surrounding FeMo-co. Changes on this time scale cannot directly be associated with catalysis. Instead, they report on changes in stability and dynamics related to equilibrium motions. The most sensitive to the presence of Fe protein on this time scale were Arg96 and His195. As a pair, they highlight changes imparted by Fe protein binding and show the specific nature of changes with the peptide containing Arg96, increasing the rate of exchange 15-fold. In contrast, the peptide containing His195 decreases its exchange rate by over 200-fold. These data are consistent with Fe protein binding triggering signal transduction that changes the protein environment surrounding the FeMo-cofactor. In some cases, dynamics increased, and in others, it decreased. Importantly, we observed a distinct time-dependence of the changes in the FeMo-co environment from as early as 10 msec (pre-steady-state) to minutes (equilibrium motions) upon Fe protein docking.

### Computational analysis of protein dynamics

Coarse-grained modeling of large-amplitude motion can be used to track protein dynamics and to follow the correlation of amino acid displacements^12,23,32,33^. Our interest in clarifying communication pathways between the two halves of the nitrogenase complex and changes in protein dynamics revealed by our HDX analysis inspired us to investigate if Fe protein controls the amplitude of motion across the MoFe protein and how the nucleotide state of Fe protein influences that motion in two distinct conformations. Therefore, we completed normal mode analysis (NMA) based on two MoFe protein-Fe protein structural models with different nucleotides bound (4WZB having β,γ-methylene MgATP and 2AFl bound by MgADP tetrafluoroaluminate). Motions within the MoFe protein tetramer and motion within and between Fe protein dimers are predominantly correlated (see Supplementary Fig. 1). However, the movement between MoFe protein and Fe protein dimers is almost exclusively anti-correlated. Nucleotide binding has a clear impact on protein motion. For example, the β,γ-methylene MgATP-bound form has greater anti-correlation within MoFe protein (darker shade of blue in specific regions of α_2_ subunits, dark red box) and more significant correlation between MoFe protein and Fe protein (darker shade of red in the regions of Fe protein, purple boxes). Additionally, the amino acid displacement is not identical when the two halves of the complex are compared (α_1_β_1_-A1B1 vs. α_2_β_2_-A2B2). This is most obvious between the corresponding regions of α_1_ (green box) and α_2_ subunits (dark red box), independently of Fe protein nucleotide status. Protein regions marked by the green/dark red boxes include the coordination sphere of the FeMo-co binding site, parts of the P-cluster environment, and the Fe protein binding interface.

Differences in the covariance matrix on a global level inspired us to look for dynamics at the active site FeMo-co that could be linked to Fe protein binding. To do this, we extracted correlation data for residues in the first coordination sphere of the MoFe-co. The amino acid displacement patterns of β,γ-methylene MgATP-bound Fe protein dynamics with respect to residues Val70, Arg96, His195, Cys275, Arg359, and His442 of the MoFe protein α_1_ subunit have a complex pattern (Supplementary Fig. 2). While the Fe protein subunit motion is predominantly anti-correlated (negative values in line plots and blue color in the structure) with the first coordination sphere residues of FeMo-co, there are several regions in the Fe protein with positive correlation (positive values in line plots and red color in the structure): Switch I, Switch II, [4Fe-4S] cluster, and the Fe protein/MoFe protein interface (around Arg140 and Lys170). Each of the regions with positive correlation has a distinct function during protein binding and catalysis^34–38^. An asymmetry of the Fe protein subunits is also observed, even though it is a homodimer (magenta asterisks). Despite identical sequences and folds, all four Fe protein subunits have distinct dynamic properties that project an asymmetry in protein dynamics.

We next extended the analysis of the Normal Mode data to include the pairs of α and β subunits in MoFe protein with respect to residues coordinating FeMo-co, specifically looking for any indication of asymmetric dynamics between structural halves of the enzyme. On a global scale, the most pronounced example is in the α subunits, where residues in α_1_(72-327) and α_2_(72-327) regions showed predominantly correlated and anti-correlated protein motion, respectively (Supplementary Fig. 3 and Supplementary Fig. 4 red boxes). Asymmetric dynamics also occur between β subunits, where yellow and green boxes in Supplementary Figs. 3 and 4 highlight regions with nucleotide-independent (β80-200) and nucleotide-dependent (β370-470) alternating protein motions. In the context of the quaternary structure, α72-327 and β370-470 regions serve as the P-cluster environment and are involved in the Fe protein/MoFe protein interface, while β80-200 regions form the α_1_β_1_/α_2_β_2_ interface. The magnitude and correlation of motion also have a nucleotide dependence, as seen by comparing the traces in complexes representing MgATP and MgADP bound states (Supplementary Fig. 3 vs 4, green boxes, and green asterisks). Interestingly, motion at the MgATP/MgADP binding site in Fe protein is independent of nucleotide type (Supplementary Figs 3 and 4, black asterisk).

### Fe protein binding changes active site dynamics

**Fig. 4:**
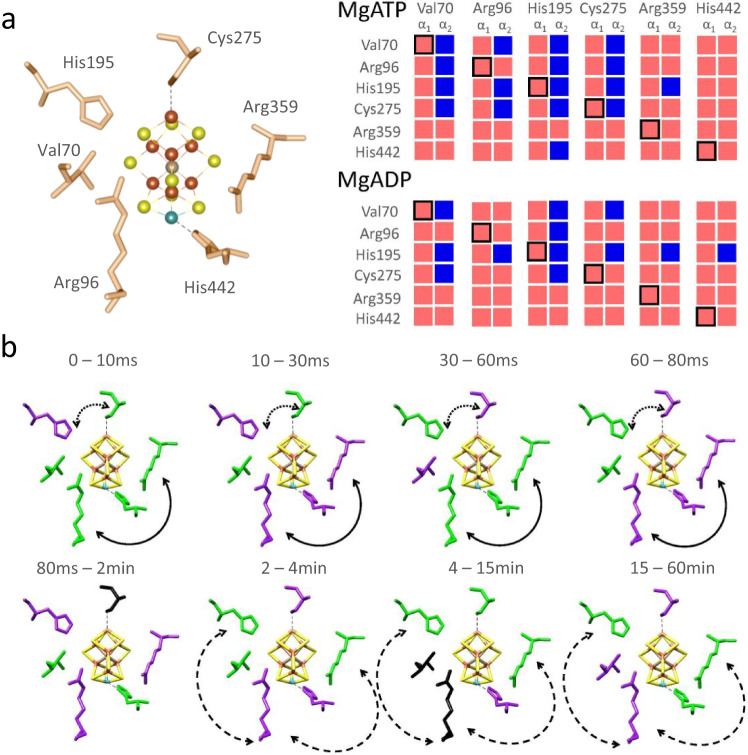
NMA and HDX of the FeMo-co active site. Protein motion relationships between individual residues/entire FeMo-co active site environment in the context of the nucleotide type bound to Fe protein. Data was extracted from NMA: **a** β,γ-methylene MgATP-bound (PDB ID: 4WZB) and MgADP tetrafluoroaluminate-bound (PDB ID: 2AFI). Red and blue blocks indicate correlated and anti-correlated residue displacement, respectively. Each pair of columns uses a different amino acid as a fiducial. Note that all residues in the α_1_ subunit are positively correlated, whereas correlation in α_2_ depends on which position in α_1_ is selected. The MgADP tetrafluoroaluminate-bound complex has more correlated motion between the selected residue in α_1_ with all of α_2_ (more red, less blue) than the ATP form. Therefore, ATP binding to Fe protein induces asymmetry in MoFe protein dynamics. **b** Time dependence of HDX rates for residues in the first coordination sphere based on peptide data. For visual clarity, the panels were simplified to show only key residues around the metal cluster. Amino acids are color-coded to indicate increased (purple) or decreased (green) rates of exchange during the indicated period after Fe protein binding to MoFe protein compared to exchange in MoFe protein alone. Residues in black were unaffected by Fe protein binding. Arg96-His442-Arg359 have correlated pre-steady-state changes in dynamics (solid black double arrow). His195-Cys275 have anti-correlated pre-steady-state changes in dynamics (dotted black double arrow). In steady-state, Arg96 motion is always anti-correlated with the motion of His195 and Arg359 and less dynamic in the presence of the Fe protein (dashed black double arrow).

The nucleotide-dependent differences in dynamics and the dynamic asymmetry between MoFe protein α_1_β_1_ and α_2_β_2_ subunits are evident in the first coordination sphere of FeMo-co (Fig. 4a). Motion within α_1_ FeMo-co is always correlated (only red boxes in the first column of each pair). In contrast, specific sites of anti-correlation emerge in the active site on the opposite side of the complex (blue boxes in the second column of each pair). The pattern is nucleotide type-dependent (except Arg359), with significant asymmetry apparent in the MgATP-bound state. The degree of the asymmetry can be best appreciated when comparing the MgATP-bound state for His195, where 5 out of 6 residues involved in the FeMo-co coordination sphere in the α_2_ subunit are anti-correlated with their counterparts in the α_1_ subunit. The second example compares Arg96 and Cys275 and illustrates how ATP hydrolysis induces the asymmetry in protein dynamics between enzyme halves. While for Arg96 and Cys275, the FeMo-co protein environment in the α_1_ subunit is positively correlated (6 out of 6 boxes are red in the first column of the pair, regardless of nucleotide type) in the α_2_ subunit, several residues highlighted by blue color is different for MgATP-bound vs MgADP-bound form. These data strongly support the half-sites cooperativity mechanism by differentiating the two active sites. NMA shows unique connectivity across the MoFe protein-Fe protein complex, suggesting that the active site FeMo-co environment is tuned by dynamics directly related to Fe protein binding and nucleotide type.

If the active site dynamics are tuned and can be modulated by Fe protein, we reasoned that biophysical evidence may be present in the HDX-MS data. Therefore, we reanalyzed our HDX data, focusing on peptides in the first coordination sphere of FeMo-co, comparing the exchange rate with and without Fe protein. Labeling residues by the direction of change (increasing or decreasing) revealed that each time point has a distinct pattern of exchange (Fig. 4b). The changes in the exchange rate across time can be either parallel (correlated) or anti-parallel (anti-correlated). For example, in pre-steady-state Arg96, Arg359, and His442 act in concordance (solid black double arrow), while His195 and Cys275 are in opposition (dotted black double arrow) for the duration of the single turnover. In addition, the timing of the change in the exchange rate seems specific for each pair/triad. Similarly, in steady-state, the exchange rate of Arg96 is always anti-correlated with His195 and Arg359 and much slower in the presence of Fe protein (dashed black double arrow). These data provide direct biophysical evidence that Fe protein binding alters the protein stability and dynamics around the FeMo-co site on a catalytically relevant timescale and at system equilibrium. Because *in vacuo* FeMo-co is a trigonal prism (electronically symmetrical) while in the enzyme, the cluster is electronically asymmetric^9,39^, the temporally explicit exchange patterns of the first coordination sphere residues suggest protein dynamics as a means for tuning the properties of the FeMo-co.

### Concluding remarks

**Fig. 5:**
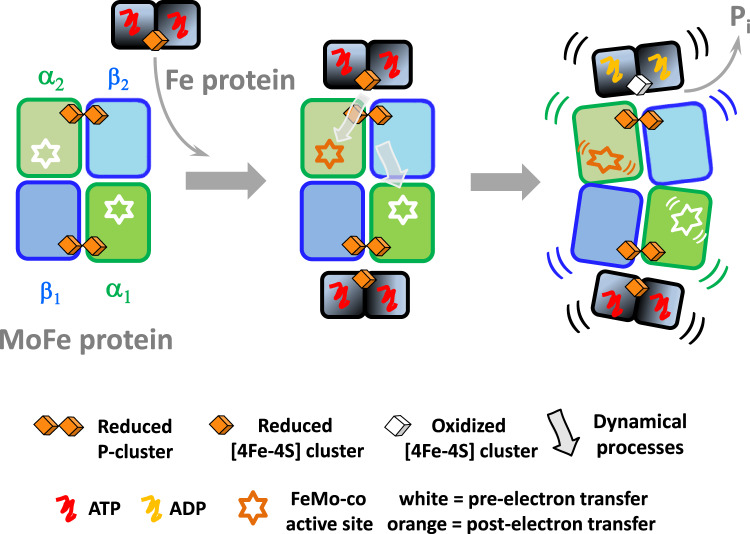
Dynamics at the active site of Mo-dependent nitrogenase are tuned by Fe protein binding in a nucleotide dependent way. Fe protein docking alters the dynamics of α and β subunits of MoFe protein in an asymmetric way which leads to a differentiation of the active sites in each half of the complex on the millisecond timescale. NMA and HDX-MS data support a mechanism in which the electronic state of the FeMo-co catalytic cofactor is tuned by altering protein dynamics in the first coordination sphere.

The biophysical and computational investigation of Fe protein-mediated electron delivery to MoFe protein has revealed long-range connectivity between Fe protein and the FeMo-co environment in the pre-steady-state and at equilibrium conditions. Our HDX measurements show that Fe protein docking initiates a wave of controlled increases and decreases in dynamics that traverses the complex from the [4Fe-4S] cluster on Fe protein through the MoFe protein P-cluster and then FeMo-co (Fig. 5). This demonstrates the long-range coupling of protein motion in the MoFe protein-Fe protein complex on a time scale relevant to catalysis. In addition, NMA displayed asymmetry in the protein environment around FeMo-co that is distinct in the two halves of MoFe protein, providing the basis for negative cooperativity. NMA also revealed that MoFe protein motion was almost exclusively anti-correlated with respect to the motion of the two Fe proteins. In addition, Fe protein binding induces long-range structural changes largely dependent on the nucleotide type. All nucleotide type-dependent changes showed asymmetry in protein motion in MoFe protein. Overall, the role of protein dynamics and tuning of the conformational landscape during catalysis is consistent with classic models of negative cooperativity. However, unlike other allosterically regulated systems, Mo-nitrogenase must undergo multiple cycles to reduce nitrogen. Using a combination of in-solution and *in-silico* tools, nucleotide-dependent tuning of the active site cofactor via protein dynamics has emerged as the likely mechanism for the coordinated reduction of dinitrogen by Mo-nitrogenase.

## Methods

### Protein purification

Nitrogenase proteins were expressed in *Azotobacter vinelandii* strains DJ995 (polyhistidine-tagged MoFe protein) and DJ884 (wild-type Fe protein). Cells were grown, and proteins were purified as previously described^40^. Proteins were concentrated using an Amicon (Millipore) anaerobic pressure concentrator with appropriate molecular weight cutoff membranes. Protein purities were assessed by SDS-PAGE (Bio-rad) analysis with Coomassie Blue stain (Thermo-Fisher) used for protein detection. Protein concentration was determined by biuret assay against a bovine serum albumin standard (Sigma).

### Hydrogen-deuterium exchange experiments

Quench flow HDX experiments were conducted using a quench flow apparatus (Kintek) housed in a Coy chamber (Coy Lab Products) under positive nitrogen pressure. Three solutions were prepared for the drive syringes: deuterated buffer (50 mM Tris, 12 mM dithionite, pD 7.4), non-deuterated buffer (50 mM Tris, 12 mM dithionite, pH 7.4), and quench solution (3% formic acid, FA). All buffers were degassed to ensure anaerobic conditions. Buffers contained 15 mM ATP and 8 mM MgCl_2_ added just before mixing. MoFe protein at 5 mg/mL (H_2_O buffer) was rapidly diluted (10-fold) during mixing with Fe protein (D_2_O buffer). Three reaction replicates were collected for MoFe protein and MoFe protein:Fe protein 1:2. For the MoFe protein-only samples, a D_2_O-based buffer without Fe protein was used. The 10-80 ms time points were quenched using the 3% FA solution. Samples were flash-frozen with liquid nitrogen immediately after quenching. Samples were subject to protease digestion with porcine pepsin (Sigma) at 0.2 mg/mL final concentration for 2 minutes before injection into the LC-MS system. Extended time points (2 min, 4 min, 15 min, 1 h) were performed on a benchtop using the protocol described previously^41,42^.

### LC-MS analysis of deuterated MoFe protein

LC-MS analysis was completed on a 1290 UPLC series chromatography stack (Agilent Technologies) coupled directly to a 6538 Q-TOF mass spectrometer (Agilent Technologies). Peptides were separated on a reverse phase column (Phenomenex Onyx Monolithic C18 column, 100 mm × 2 mm) at 1 °C using a flow rate of 500 µL/min under the following conditions: 0.0-1.0 min, 5% B; 1.0-11.0 min, 5-45% B; 11.1-11.8 min, 95% B; 11.8-11.9 min, 5% B. Solvent A contained 0.1% formic acid in water (Thermo-Fisher) while solvent B contained 0.1% formic acid in acetonitrile (Thermo-Fisher). Data were acquired at 2 Hz over a scan range 50-1700 m/z in positive mode. Electrospray settings: nebulizer 3.7 bar, drying gas 8.0 L/min., drying temperature 350 °C, and capillary voltage 3.5 kV. MS/MS of non-deuterated protein digests, specifying a selection window of 4 m/z, was used to generate peptide sequence tags. Data processing was carried out in Agilent MassHunter Qualitative Analysis version 6.0 (Agilent Technologies). Non-deuterated peptide identification was performed using the peptide analysis worksheet for single-level MS (PAWs, ProteoMetrics LLC.) and Peptide Shaker (CompOmics, version 1.16.15) for MS/MS^43^.

### HDX-MS analysis

Deuterium uptake was determined by monitoring shifts in the centroid of peptide isotopic distributions using HDExaminer (Sierra Analytics Inc., Version 2.4.1). Measured values were used to generate uptake curves to compare deuterium incorporation in all conditions. 37 peptides from the alpha subunit and 26 from the beta subunit showed a high confidence deuterium incorporation score (>0.70). The percent deuterium incorporated (%D) was calculated using the number of deuterium incorporated after 24 h. Butterfly plots were developed in Microsoft Excel using %D at each time point for all identified peptides (Supplementary Data 1). All molecular graphics were generated in UCSF Chimera (version 1.16)^44^.

### Normal mode analysis

A normal mode analysis was carried out using the anisotropic Gaussian network model to characterize the long-timescale dynamics of the nitrogenase complex. The nitrogenase complex was represented by beads centered at the position of the α-carbons. Two beads are connected with a harmonic spring if they are within a 12 Å cutoff distance and not connected otherwise. The correlations between the fluctuations of the protein residues were analyzed in terms of the normalized covariance matrix of the inter-residue displacements. Calculations were performed on the crystal structure of the nitrogenase complex between Fe protein and MoFe protein from *A. vinelandii* with both two ATP analogs (β,γ-methylene MgATP) (PDB ID: 4WZB^29^), and two MgADP (PDB ID: 2AFI^29^,) bound to each Fe protein. The presence of the various cofactors (iron/sulfur clusters, nucleosides, and homocitrate) was considered by adding beads as follows. (i) For the Fe protein cubane and the P-cluster, a bead was placed at the geometric center of each cluster. (ii) For the FeMo-co, two beads were added: one at the center of the Fe1, Fe2, Fe3, and Fe4 tetrahedron and the other at the center of the Fe5, Fe6, Fe7, and Mo tetrahedron. (iii) The homocitrate was described by a bead at its geometric center. (iv) ATP and ADP were modeled with three beads at the center of the adenine ring, the ribose ring, and the phosphate groups. As previously discussed, this representation of the nitrogenase complex accurately reproduces the relative magnitude of the experimental X-ray beta factors, giving confidence that the approach can describe the overall large amplitude motions of the nitrogenase complex^12,32^.

### Reporting summary

Further information on research design is available in the Nature Portfolio Reporting Summary linked to this article.

### Supplementary information

Supplementary_information

Description of Additional Supplementary Files

Supplementary Data 1

Reporting Summary

## Data Availability

All the data supporting this article have been included in the main text and the Supplementary Information.
