# Supplementary material for: Fe protein docking transduces conformational changes to MoFe nitrogenase active site in a nucleotide-dependent manner
Source: Commun Chem. 2023 Nov 18;6:254. doi: 10.1038/s42004-023-01046-6 (PMC10657360; doi:10.1038/s42004-023-01046-6)
Supplement: Supplementary file 1 — Supplementary_information [file 42004_2023_1046_MOESM1_ESM.pdf]

Supplementary Material to:

Fe protein docking transduces conformational changes to MoFe nitrogenase active site in a nucleotide-dependent manner

Monika Tokmina-Lukaszewska[1], Qi Huang[2], Luke Berry[1], Hayden Kallas[3], John W. Peters[4], Lance C. Seefeldt[3], Simone Raugei[2] and Brian Bothner*[1].

1. Department of Chemistry and Biochemistry, Montana State University, Bozeman, MT United States of America.

2. Physical and Computational Sciences Directorate, Pacific Northwest National Laboratory, Richland, WA United States of America.

3. Department of Chemistry and Biochemistry, Utah State University, Logan, UT United States of America.

4. Institute of Biological Chemistry, The University of Oklahoma, Norman, OK United States of America.

*corresponding author: bbothner@montana.edu

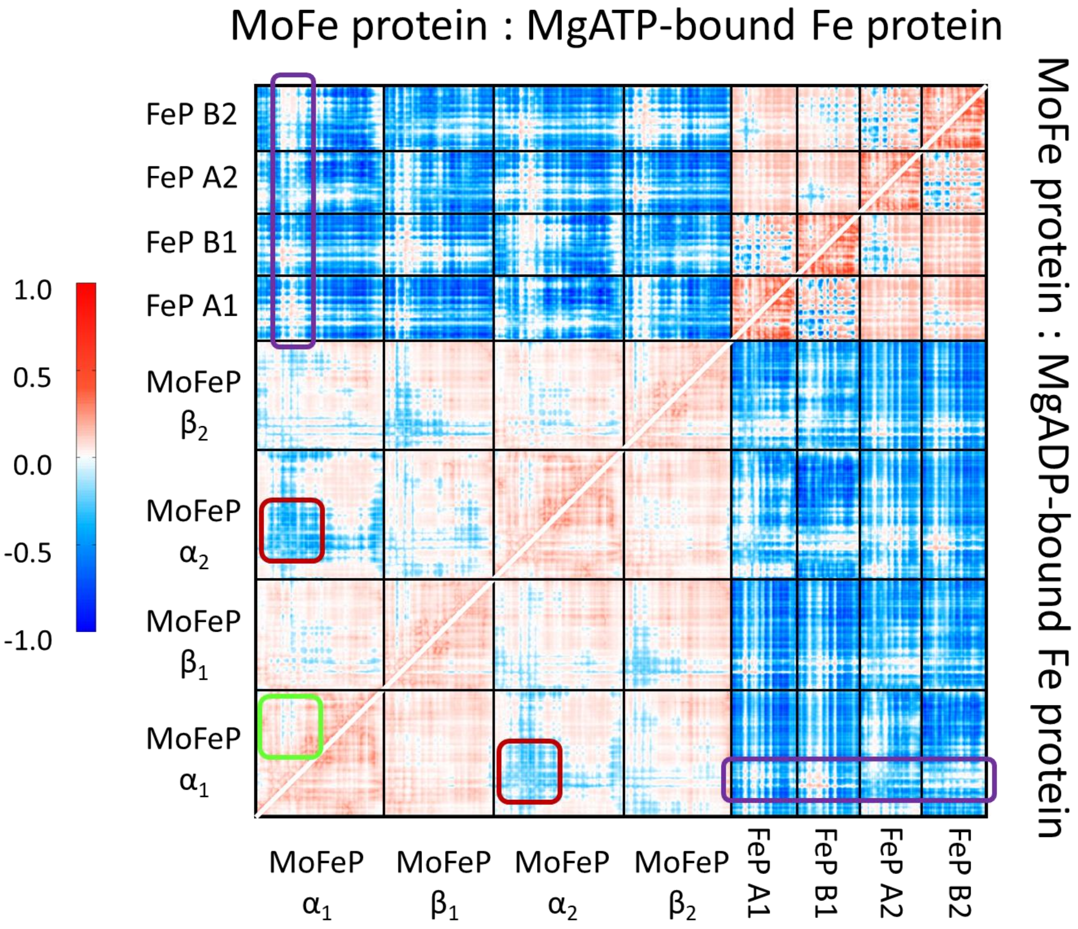

Supplementary Figure 1. Covariance matrix showing the correlation of amino acid displacement in the MoFe protein and Fe protein from NMA.
Upper diagonal: MoFe protein:Fe protein β,γ-methylene MgATP complex (PDB ID: 4WZB). **Lower diagonal:** MoFe protein:Fe protein tetrafluoroaluminate MgADP complex (PDB ID: 2AFI). The axes represent the amino acid sequence for each protein subunit, with each row and column containing correlative data for a single residue with all others. Red and blue indicate correlated (positive values) and anti-correlated (negative values) residue displacement, respectively. Motion within the MoFe protein tetramer is generally correlated and predominantly anti-correlated with Fe protein dimers (mostly red for MoFe protein and all blue for the four Fe protein subunits). Examples of the anti-correlated protein motions are shown in: green box (α_1 subunit of ATP-bound form), dark red boxes (α_2 subunits of ATP- and ADP-bound forms) and purple boxes (A1B1/A2B2 subunits of ATP- and ADP-bound forms).

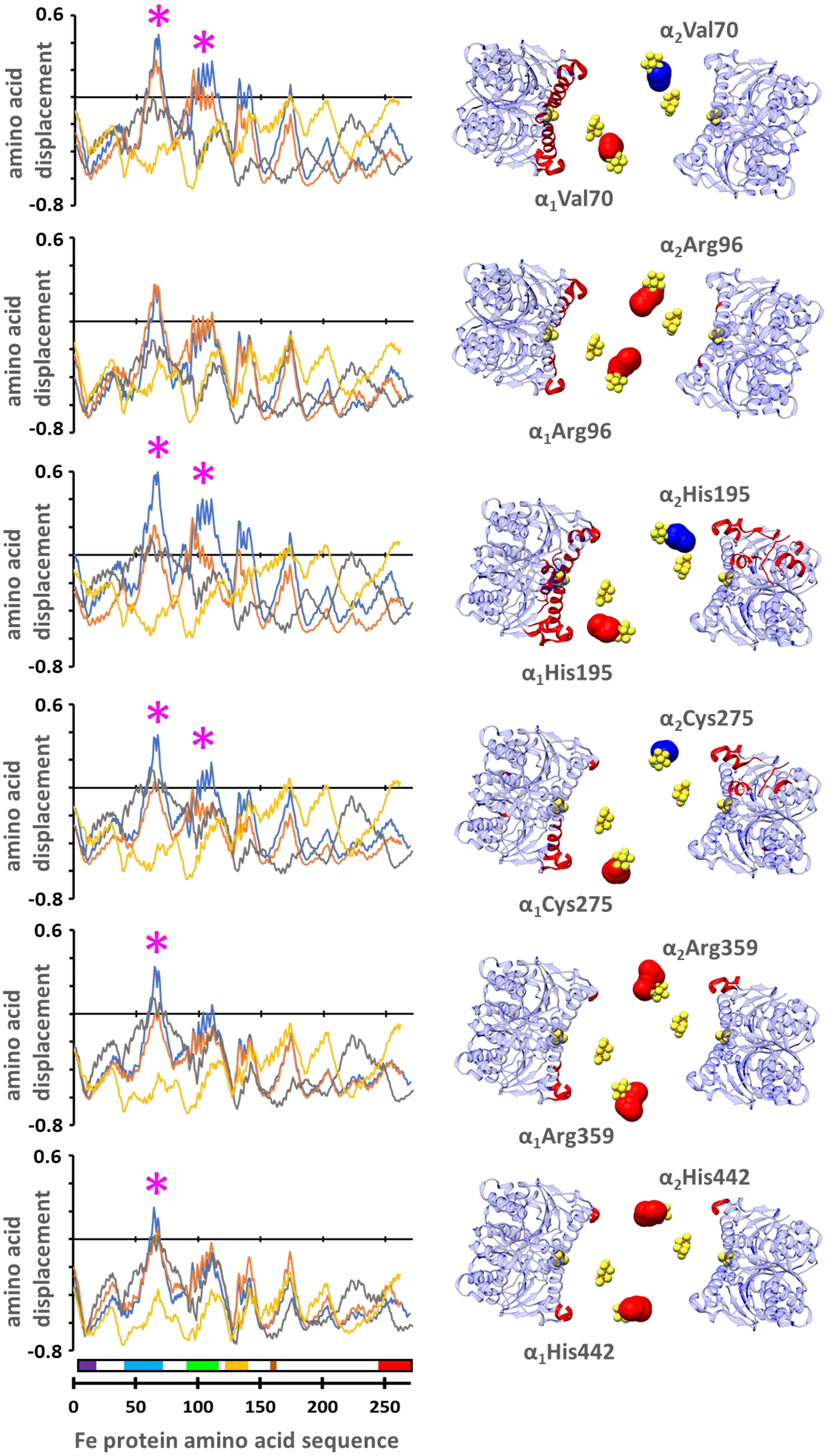
0.6
-0.8
amino acid displacement
*
α2Val70
α1Val70
α2Arg96
α1Arg96
α2His195
α1His195
α2Cys275
α1Cys275
α2Arg359
α1Arg359
α2His442
α1His442
0
50
100
150
200
250
Fe protein amino acid sequence

Supplementary Figure 2. Normal mode analysis shows connectivity between Fe protein and the FeMo-co active site.

Line plots display protein motion for the Fe protein with respect to residues in the first coordination sphere of the FeMo-co (α_1 subunit: Val70, Arg96, His195, Cys275, Arg359 and His442). Comparison is based on the Fe protein in the MoFe protein:Fe protein β,γ-methylene MgATP complex. Light blue, orange, gray, and yellow lines represent residues in A1, B1, A2, and B2 Fe protein subunits, respectively. Colored bars above x axis correspond to following regions of Fe protein amino acid sequence going from N- to C-terminus: Nucleotide-binding site (purple), Switch I (blue), [4Fe-4S] cluster (green), Switch II (yellow), Lys170 (orange) and C-terminal (red). The motion is largely anti-correlated (negative values) and shows distinct patterns between subunits in the same dimer (A1 vs B1), as well as between Fe proteins (A1B1 vs A2B2). Fe protein regions that are correlated (positive values) with residues in the first coordination sphere of FeMo-co have distinct functional roles and include MgATP binding, Switch I, Switch II, and the [4Fe-4S] cluster cofactor. Structural models display the correlation patterns with blue showing anti-correlated and red correlated motion. NMA also shows that Fe protein binding differentiates the FeMo-co sites. For example, Val70, His195, and Cys275 are anti-correlated in α_1 vs α_2, while Arg96, Arg359, and His442 are correlated. Magenta asterisks highlight differences in magnitude of protein motion within homodimeric Fe protein which are primarily observed in Switch I region and [4Fe-4S] cluster environment.

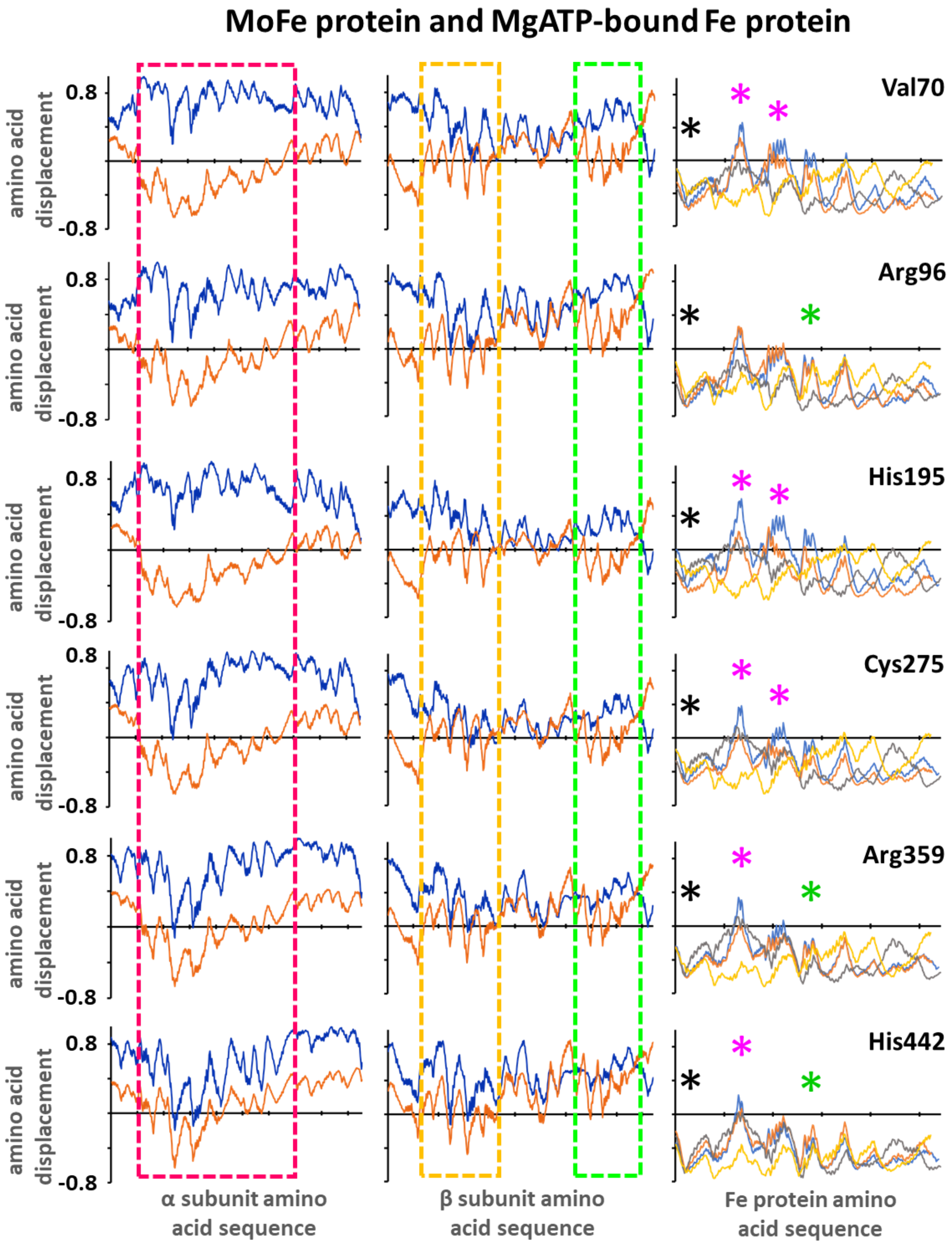

MoFe protein and MgATP-bound Fe protein
amino acid displacement
0.8
-0.8
Val70
Arg96
His195
Cys275
Arg359
His442
*
α subunit amino acid sequence
β subunit amino acid sequence
Fe protein amino acid sequence

Supplementary Figure 3. Protein motion correlated with amino acids in the first coordination sphere of the FeMo-co from MgATP-bound form.
Amino acid displacement calculated by NMA across the MoFe protein:Fe protein complex is dependent on the Fe protein nucleotide state. Protein motion patterns of selected residues in the FeMo-co environment (α_1 subunit: Val70, Arg96, His195, Cys275, Arg359 and His442) from the MoFe protein:Fe protein β,γ-methylene MgATPcomplex (PDB ID: 4WZB). In the first and second column of each panel: light blue and orange lines correspond to $\alpha_1\beta_1$ and $\alpha_2\beta_2$ subunits, respectively. In the third column of each panel: light blue, orange, gray, and yellow lines correspond to residues in A1, B1, A2 and B2 subunits of Fe protein, respectively. The red box highlights a region of asymmetric protein motion in the α subunits (α72-327). Yellow and green boxes highlight the β subunit regions with alternating nucleotide type independent (β80-200) and nucleotide type-dependent (β370-470) motion, respectively. Green asterisks show regions of nucleotide type induced asymmetric protein motion in Fe protein (near Lys170 residue). Magenta asterisks highlight differences in magnitude of protein motion within homodimeric Fe protein (Switch I region and [4Fe-4S] cluster environment). Black asterisks highlight the Fe protein nucleotide binding site and show no difference with respect to MgATP or MgADP. The difference in dynamics between pairs of subunits indicates that the complex is dynamically quasi-equivalence of MoFe protein subunits (blue vs orange) and Fe protein subunits (blue vs orange vs gray vs yellow).

MoFe protein and MgADP-bound Fe protein

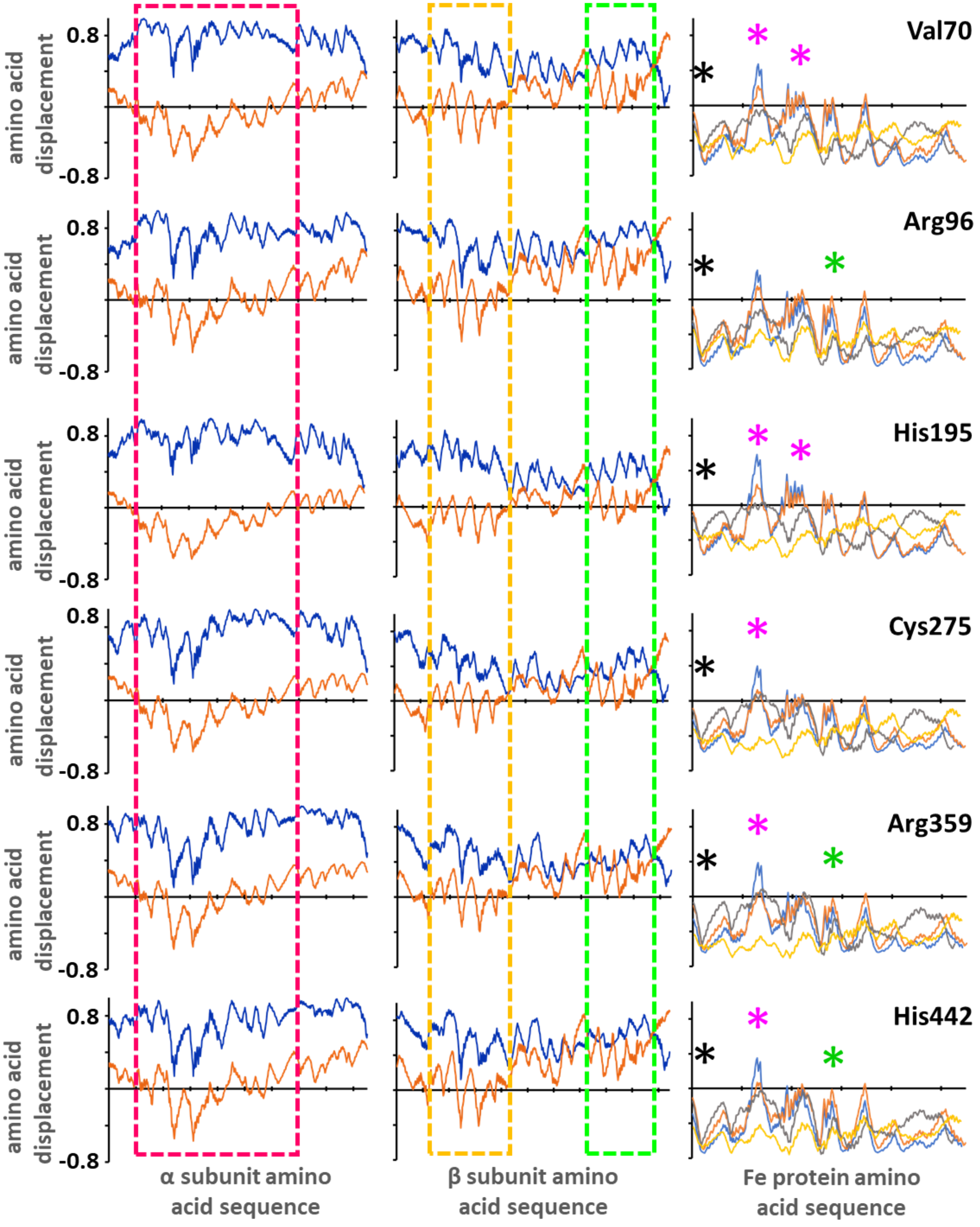

Supplementary Figure 4. Protein motion correlated with amino acids in the first coordination sphere of the FeMo-co from MgADP-bound form.

Amino acid displacement calculated by NMA across the MoFe protein:Fe protein complex is dependent on the Fe protein nucleotide state. Protein motion patterns of selected residues in the FeMo-co environment (α_1 subunit: Val70, Arg96, His195, Cys275, Arg359 and His442) from the MoFe protein:Fe protein tetrafluoroaluminate MgADP complex (PDB ID: 2AFI). In the first and second column of each panel: light blue and orange lines correspond to $\alpha_1\beta_1$ and $\alpha_2\beta_2$ subunits, respectively. In the third column of each panel: light blue, orange, gray, and yellow lines correspond to residues in A1, B1, A2 and B2 subunits of Fe protein, respectively. The red box highlights a region of asymmetric protein motion in the α subunits (α72-327). Yellow and green boxes highlight the β subunit regions with alternating nucleotide type independent (β80-200) and nucleotide type-dependent (β370-470) motion, respectively. Green asterisks show regions of nucleotide type induced asymmetric protein motion in Fe protein (near Lys170 residue). Magenta asterisks highlight differences in magnitude of protein motion within homodimeric Fe protein (Switch I region and [4Fe-4S] cluster environment). Black asterisks highlight the Fe protein nucleotide binding site and show no difference with respect to MgATP or MgADP. The difference in dynamics between pairs of subunits indicates that the complex is dynamically quasi-equivalence of MoFe protein subunits (blue vs orange) and Fe protein subunits (blue vs orange vs gray vs yellow).
