# Supplementary material for: Fe protein docking transduces conformational changes to MoFe nitrogenase active site in a nucleotide-dependent manner
Source: Commun Chem. 2023 Nov 18;6:254. doi: 10.1038/s42004-023-01046-6 (PMC10657360; doi:10.1038/s42004-023-01046-6)
Supplement: Supplementary file 2 — Description of Additional Supplementary Files [file 42004_2023_1046_MOESM2_ESM.pdf]

Description of Additional Supplementary Files

File name: Supplementary Data 1

Description: HDX dataset for MoFe protein alone and in the presence of Fe protein
